# Persistent megalocystic ovaries after ovarian hyperstimulation syndrome in a postpartum patient with polycystic ovarian syndrome: a case report

**DOI:** 10.1186/s13048-018-0451-7

**Published:** 2018-09-10

**Authors:** Jinghua Shi, Xinyu Ren, Qinjie Tian, Aijun Sun, Rong Chen

**Affiliations:** 1 Department of Obstetrics and Gynecology, Peking Union Medical College Hospital (PUMCH), Peking Union Medical College, Chinese Academy of Medical Science, Beijing, 100730 People’s Republic of China; 2 Department of Pathology, Peking Union Medical College Hospital (PUMCH), Peking Union Medical College, Chinese Academy of Medical Science, Beijing, People’s Republic of China

**Keywords:** Persistent megalocystic ovaries, Ovarian hyperstimulation syndrome, Polycystic ovarian syndrome, Ovarian torsion

## Abstract

**Background:**

Ovary enlargement is common in controlled ovarian stimulation, which could continue several months during a successful pregnancy. However, persistent megalocystic ovaries 3 years after ovarian hyperstimulation syndrome (OHSS) were rare. Here we will present you the case and treatment as well as discuss the probable etiology.

**Case presentation:**

A 34-year-old woman with polycystic ovarian syndrome (PCOS) and a history of infertility presented to the Department of Obstetrics and Gynecology at Peking Union Medical College Hospital with abdominal pain and persistently enlarged ovaries 36 months after OHSS. Enlarged ovaries were evaluated with ultrasonography and serum tests. Diagnostic laparoscopic surgery with detorsion and drainage followed by GnRHa treatment was performed. Symptoms and ovarian size evaluated by vaginal ultrasound were the main outcome measures. The patient was discharged from the hospital 5 days after surgery without any remarkable complications. Both ovaries recovered to almost normal after a monthly injection of GnRHa for 3 months.

**Conclusions:**

Ovarian enlargement may persist for a long time in patients with severe OHSS even after sex hormone levels and ovarian functions return to normal. Long term follow-up is necessary and ovarian torsion should be suspected when accompanied by abdominal pain. Acupuncture plus GnRHa treatment may be an effective way for these cases.

## Background

Ovarian hyperstimulation syndrome (OHSS) is an excessive response to controlled ovarian hyperstimulation during treatment cycles used for assisted reproduction technology (ART). Moderate OHSS occurs during 3–6% of all cycles, whereas the severe form occurs during 0.1% of all cycles [1]. For women at high risk for OHSS, this incidence approaches 20% [2]. Conditions associated with a higher risk of OHSS include young age, low body mass index, polycystic ovarian syndrome (PCOS), higher doses of exogenous gonadotropins, high absolute or increased rates of serum estradiol (E_2_) levels, and previous OHSS [3].

Early-onset OHSS occurs within 9 days after oocyte retrieval and will typically resolve within 7 days if no pregnancy occurs; however, late-onset OHSS appears 10 days after oocyte retrieval [4]. When pregnancy is maintained, symptoms of luteal cysts usually resolve gradually within 1–2 months and rarely persist until the 5th month of gestation [5].

We describe a case of persistent bilateral megalocystic ovaries in a patient with PCOS who became pregnant following in vitro fertilization (IVF). Large ovarian cysts persisted throughout the pregnancy and more than 2 years after delivery. To our knowledge, this is the first case of enlarged ovaries that persisted 36 months after OHSS.

## Case presentation

**Fig. 1:**
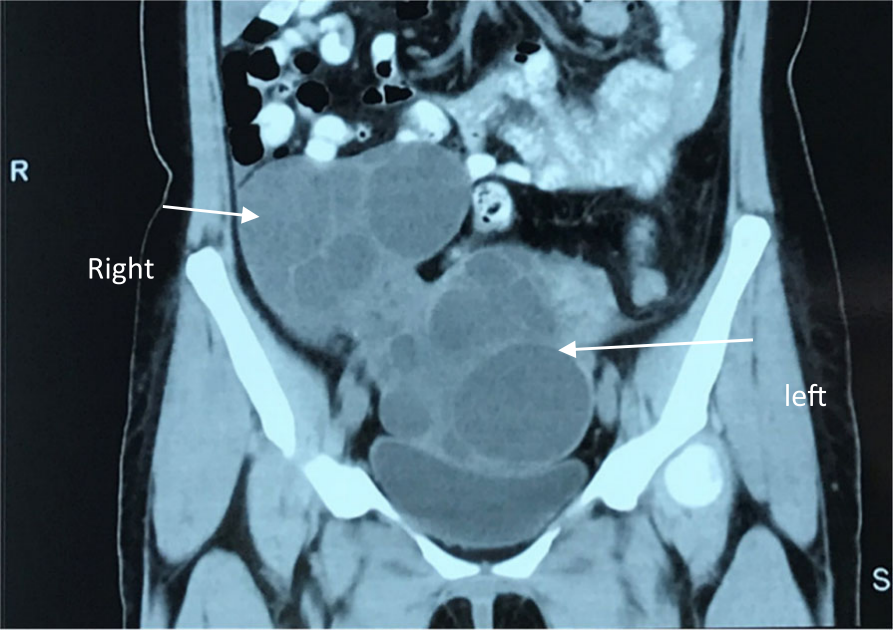
Computed tomography (CT) scan of the patient. CT showed bilateral enlarged ovaries with multiple septations in abdomen and pelvis

A 34-year-old woman (gravida 4, para 1, abort 3) presented to our clinic for pelvic pain and enlarged ovaries at PUMCH (Peking Union Medical College Hospital) with a 5-day history of left lower quadrant abdominal pain. The pain was atypical, without nausea, vomiting, dysuria, or diarrhea. Her last menstrual period was 2 weeks prior to presentation. There were palpable, cystic, solid masses on both sides in the lower quadrant. Laboratory tests revealed a white blood cell count of 22.9 × 10^9^/L, granulocyte rate of 80.6%, and a normal β-human chorionic gonadotropin (β-hCG) level. She had a transient fever of 37.9 °C; therefore, antibiotics was administered for 4 days. When she came to our hospital, pelvic pain was relieved. Ultrasound imaging and computed tomography (Fig. 1) revealed that both the ovaries were enlarged (≥10 cm) with multiple follicles inside. Serum hormone levels were normal: follicle-stimulating hormone (FSH), 2.38 IU/L; E_2_, 46.85 pg/mL; progesterone (P), 0.35 ng/mL; testosterone (T), 0.54 ng/mL; luteinizing hormone (LH), < 0.2 IU/L; prolactin (PRL), 7.44 ng/mL.Dehydroepiandrosterone (DHEA), 497.5 μg/dL and 24-h urinary-free cortisol (UFC), 165.24 μg were slightly higher than normal. Adrenal ultrasound, serum thyroid-stimulating hormone (TSH)/free thyroxine (FT4), thyroxine (T4) and hypothalamic-pituitary magnetic resonance imaging revealed no abnormality. The concentration of tumor marker CA125 was 365.7 U/mL; therefore, a malignant tumor could not be excluded.

Before presentation, she was diagnosed with PCOS and underwent several attempts of ovulation induction and intrauterine insemination. After these failed, she underwent IVF with Marvelon (N.V. Organon, Oss, The Netherlands) and GnRHa stimulation. A combined estrogen and progesterone pill (Marvelon; N.V. Organon) was administered from day 5 of the previous cycle, and 1.2 mg triptorelin embonate (Diphereline; Ipsen Pharma Biotech, France) was injected intramuscularly on day 16 of taking Marvelon. Stimulation with recombinant follicle-stimulating hormone (Puregon; N.V.Organon) was started subcutaneously after 16 days’ down-regulation. Human chorionic gonadotropin (HCG) 5,000 IU was injected when the maxium follicle diameter reached 20 mm. The IVF procedure was performed at another center; therefore, details of estrogen and follicle development could not be traced. Transvaginal oocyte retrieval was uneventful and yielded 24 mature oocytes. Two blastocysts were transferred 4 days later. The patient had severe OHSS 10 days after oocyte retrieval, for which paracentesis was performed three times, with an average of 1,500 mL abdominal effusion drained each time. She was also suspected to have vein thrombosis of the right lower limbs. The patient became pregnant, and the follow-up was performed at another center. Throughout her perinatal examinations, both the ovaries did not become smaller. The patient delivered a healthy newborn via cesarean at term, a biopsy of the enlarged ovary was performed with benign pathology. No intervention was performed due to the expectation that the hyperstimulated ovaries would shrink during the postpartum period, at the same time she was concerned about the side-effects of those medicines in lactation. Her menstrual period resumed 14 months after delivery, and the child was weaned from breastfeeding at 24 months. However, the size of both the ovaries were still not reduced by then. Three months of oral contraceptives (Marvelon; N.V. Organon) were prescribed.

**Fig. 2:**
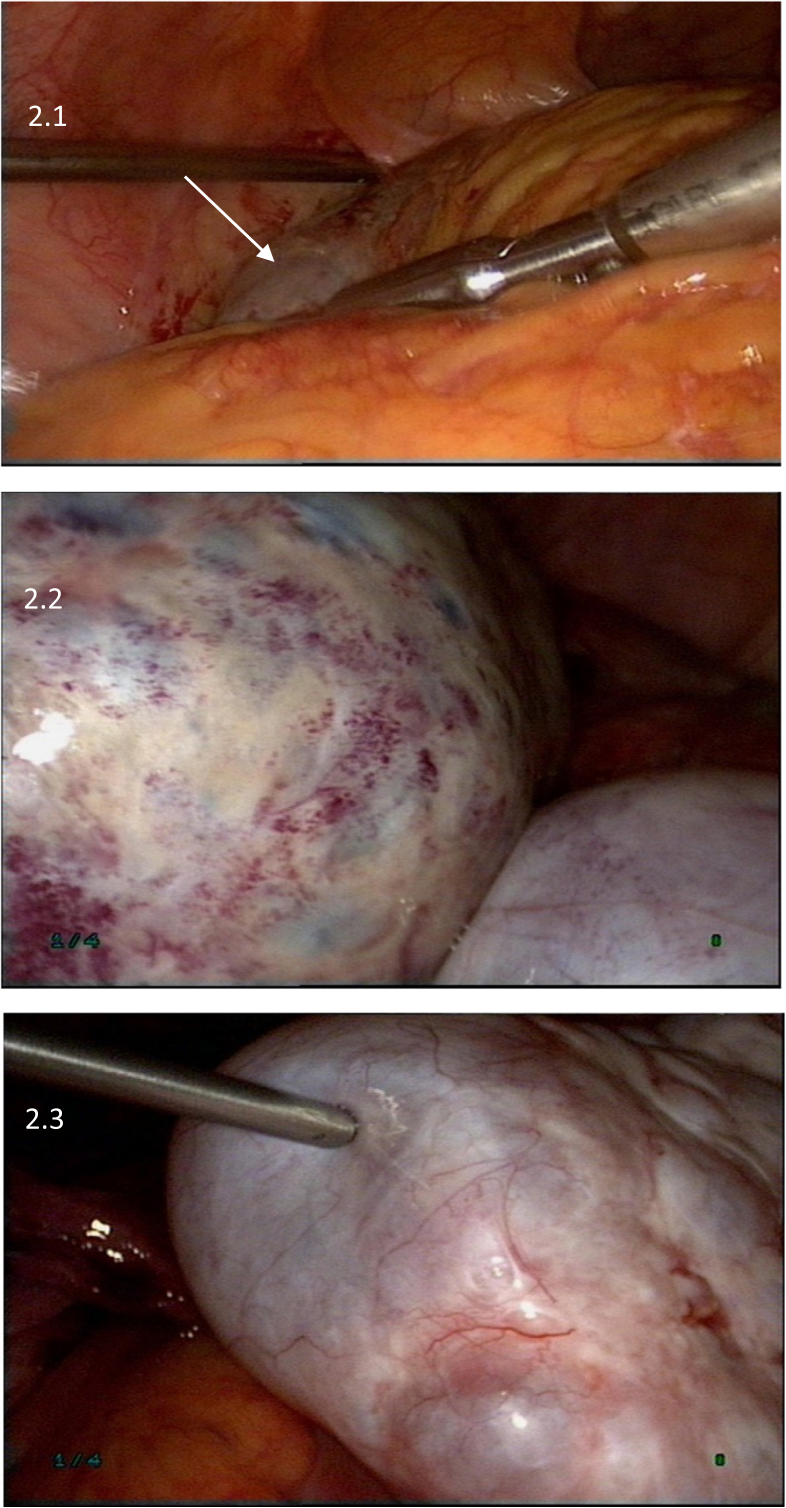
Appearance of ovaries in surgery. Laparoscopic surgery showed the omentum majus adhering to the left ovary (arrow) which was ischemic caused by ovarian torsion with a maximum diameter of 10+ cm (2.1). Both adnexae formed “kissing ovaries” (2.2).The right ovary, measuring about 10 cm was multinodulated with yellow serous fluid (2.3)

**Fig. 3:**
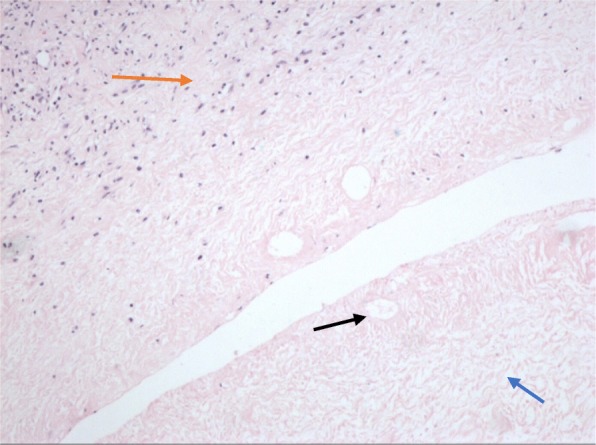
Histology of the tissue (× 100). Detailed legends: the upper left part of the figure showed the survival of the ovarian cortex with short-fusiform and wavy cells (), while the lower right coagulation necrosis, uniform red staining and nucleus lysis (), but the organizational structure could be seen vaguely. Necrotic cavities () in the necrosis were the remnants of small blood vessels

After admission, she underwent laparoscopic surgery to determine the cause of the persistent enlarged ovaries as well as pain. During laparoscopy, we found a large, torsed, congestive left ovary and a torsed, congestive, ipsilateral fallopian tube. The contralateral adnexa were enlarged but had a normal color. Both ovaries measured approximately 10 × 12 cm, kiss-forming, were closely stuck together. There were minimal ascites in the abdominal cavity. The omentum majus was adhered to and laid over the left ovary. Laparoscopic detorsion followed by left ovary biopsy and bilateral ovarian acupuncture were performed (Fig. 2). Histopathological examination (Fig. 3) revealed localized congestion and necrosis of the ovary that underwent biopsy, with no associated lesions. Ovary puncture liquid showed elevated E_2_ (2,078 pg/mL) and decreased FSH (0.3 IU/L) and LH (< 0.2 IU/L). The postoperative course was uneventful.

The patient was discharged the following week and received GnRHa 3.75 mg for 3 months. The ovaries shrank somewhat during the first month (left ovary, 5.8 × 5.1 cm; right ovary, 9.3 × 6.3 cm). Four months after surgery, she underwent an ultrasound scan that found slightly enlarged ovaries with multiple follicles (left ovary, 6.5 × 4.7 cm; right ovary, 4.1 × 3.0 cm). She did not feel any discomfort; therefore, she was advised to return 6 months later with no further treatment.

## Discussion and conclusions

### Mechanism of ovarian enlargement

Ovarian enlargement secondary to hyperstimulation is common, especially for PCOS with enlarged ovaries at baseline. According to the Rotterdam criteria, PCOS itself is defined as enlarged ovary with a follicle number of ≥12 per ovary and/or an ovarian volume of > 10 mL in at least one ovary [6]. During ovulation induction, multiple small follicles grow under hormone stimulation and hCG stimulates the ovaries to continue to grow. Pregnant women are continuously exposed to endogenous hCG. Most ovarian enlargement with multiple follicular and lutein cysts persists for a longer period (until the second trimester), because hCG starts to decline to 40,000 IU/L at 20 weeks of gestation. Renal and hepatic functions, which are normal in ordinary controlled ovary hyperstimulation, might altered in sever OHSS and disturb the hormone metabolism [7]. There are many other benign or malignant tumors that need to be differentiated, such as hyperreactio luteinalis, theca lutein cysts, teratoma, endometriosis cyst, mucinous cystadenoma, and others [8]. However, cases of persistent megalocystic ovaries existing for a long time after IVF are rarely reported, and the possible mechanism is unknown.

### Indication for exploratory laparoscopy

Ovarian torsion, the fifth most common gynecological emergency, is defined as the partial or complete rotation of the ovarian vascular pedicle. It causes obstruction of the venous outflow and arterial inflow. It is uncommon for an ovary of normal size to become twisted, but enlarged ovaries are prone to torsion. Pregnant patients are reported to have a 1% increased risk of ovarian torsion compared to nonpregnant patients. The incidence of ovarian torsion after IVF treatment is rare, ranging from 0.08 to 0.13% [9]. However, when torsion occurs during pregnancy, symptoms are less atypical, and the decision regarding whether to perform exploratory laparoscopy is difficult. Symptoms like lower abdominal pain, tenderness with a palpable mass, nausea, vomiting, low-grade fever, and leukocytosis are not significant. Ovarian enlargement secondary to IVF is usually bilateral, but torsion rarely occurs on both sides. Therefore, chronic partial torsion might be missed in some cases when clinical follow-up is used rather than surgery.

The best treatment for ovarian torsion is early diagnosis and prompt surgical intervention. The ovary is untwisted to restore the blood supply, and color changes are observed for 10–15 min. Cystectomy or oophorectomy of the torsed ovary is based on the degree of ischemia and necrosis. Laparoscopic surgery is preferred [10], because it results in less postoperative pain, shorter hospital stays, reduced adhesion formation, and a faster return to normal diet and work. Losing ovary due to a delayed diagnosis in an infertile woman is the worst consequence [11].

### Treatment of persistent megalocystic ovaries

Ling et al. [12] reported a case of persistent megalocystic ovaries during cesarean delivery for a PCOS patient with a pregnancy induced by IVF. The megalocystic ovaries persisted after delivery; therefore, the patient underwent surgery during which biopsies were performed for both ovaries. The histopathological results indicated follicular cysts. Alptekin et al. [13] reported large ovaries during cesarean delivery for a patient without OHSS but who had undergone IVF. However, the uterus and ovaries returned to normal 4 weeks later. Ours is the longest reported case of megalocystic ovaries. There has been no report of or guidance regarding the treatment of persistent megalocystic ovaries lasting such a long time in IVF patients.

The team finally chose the puncture and GnRH agonist protocol based on the high level of hormones in the cystic fluid. Although these hormones were not increased in the blood, the E_2_ levels were quite high in the follicles. The cumulative effect of such high hormone levels in so many cysts might have an important role in maintaining enlarged ovaries. GnRH agonists [14] have decreased luteotropic effects and alter the expressions of VEGF, VEGF receptor-1, and VEGF receptor-2; they have also been shown to be effective for preventing OHSS in high-risk patients. Both ovaries shrank somewhat after surgery, and they gradually returned to normal size during follow-up after three doses of GnRH agonists, which validated our method.

In conclusion, hyperstimulated, enlarged ovaries and their complications could be persistent during and even after pregnancy when IVF is involved. The risks of malignancy and torsion must be kept in mind, but should not lead to unnecessary surgery. Long-term follow-up of IVF patients is recommended.

## Abbreviations

ART: Assisted reproduction technology
DHEA: Dehydroepiandrosterone
E2: Estradiol
FSH: Follicle-stimulating hormone
FT4: Free thyroxine
GnRHa: Gonadotropin-releasing hormone agonist
IVF: In vitro fertilization
LH: Luteinizing hormone
OHSS: Ovarian hyperstimulation syndrome
P: Progesterone
PCOS: Polycystic ovarian syndrome
PRL: Prolactin
T: Testosterone
T4: Thyroxine
TSH: Thyroid-stimulating hormone
UFC: Urinary-free cortisol
VEGF: Vascular endothelial growth factor
β-hCG: Human chorionic gonadotropin
